# Supplementary material for: Growing Human Hepatocellular Tumors Undergo a Global Metabolic Reprogramming
Source: Cancers (Basel). 2021 Apr 20;13(8):1980. doi: 10.3390/cancers13081980 (PMC8074141; doi:10.3390/cancers13081980)
Supplement: Supplementary file 1 [file cancers-13-01980-s001.zip › cancers-1158659-supplementary.pdf]

Growing Human Hepatocellular Tumors Undergo a Global Metabolic Reprogramming

Fangrong Zhang, Yingchao Wang, Geng Chen, Zhenli Li, Xiaohua Xing, Csilla Putz-Bankuti, Rudolf E. Stauber, Xiaolong Liu and Tobias Madl

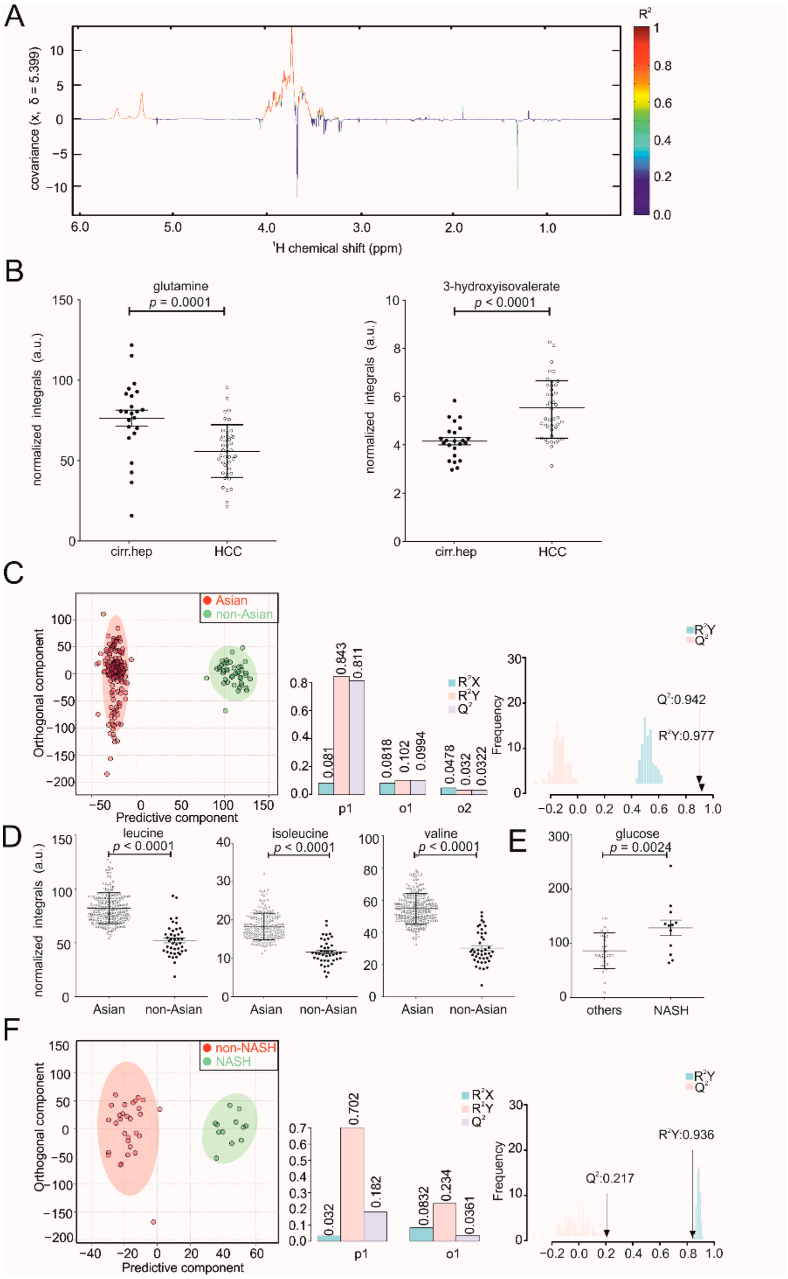

Figure S1. NMR metabolomics analysis of serum/plasma samples. (**A**) Statistical total correlation spectroscopy (STOCSY) analysis of NMR signals at 5.399 ppm. (**B**) Statistical analysis of individual metabolites in serum samples of European cohort. (**C**) O-PLS-DA plot of HCC serum/plasma samples, including cross validation (Asian vs. non-Asian). (**D**) Statistical analysis of individual metabolites in serum/plasma samples (Asian vs. non-Asian). (**E**) Statistical analysis of individual metabolites in serum/plasma samples (others vs. NASH). (**F**) O-PLS-DA plot of HCC serum/plasma samples (European cohort), including cross validation (others vs. NASH).

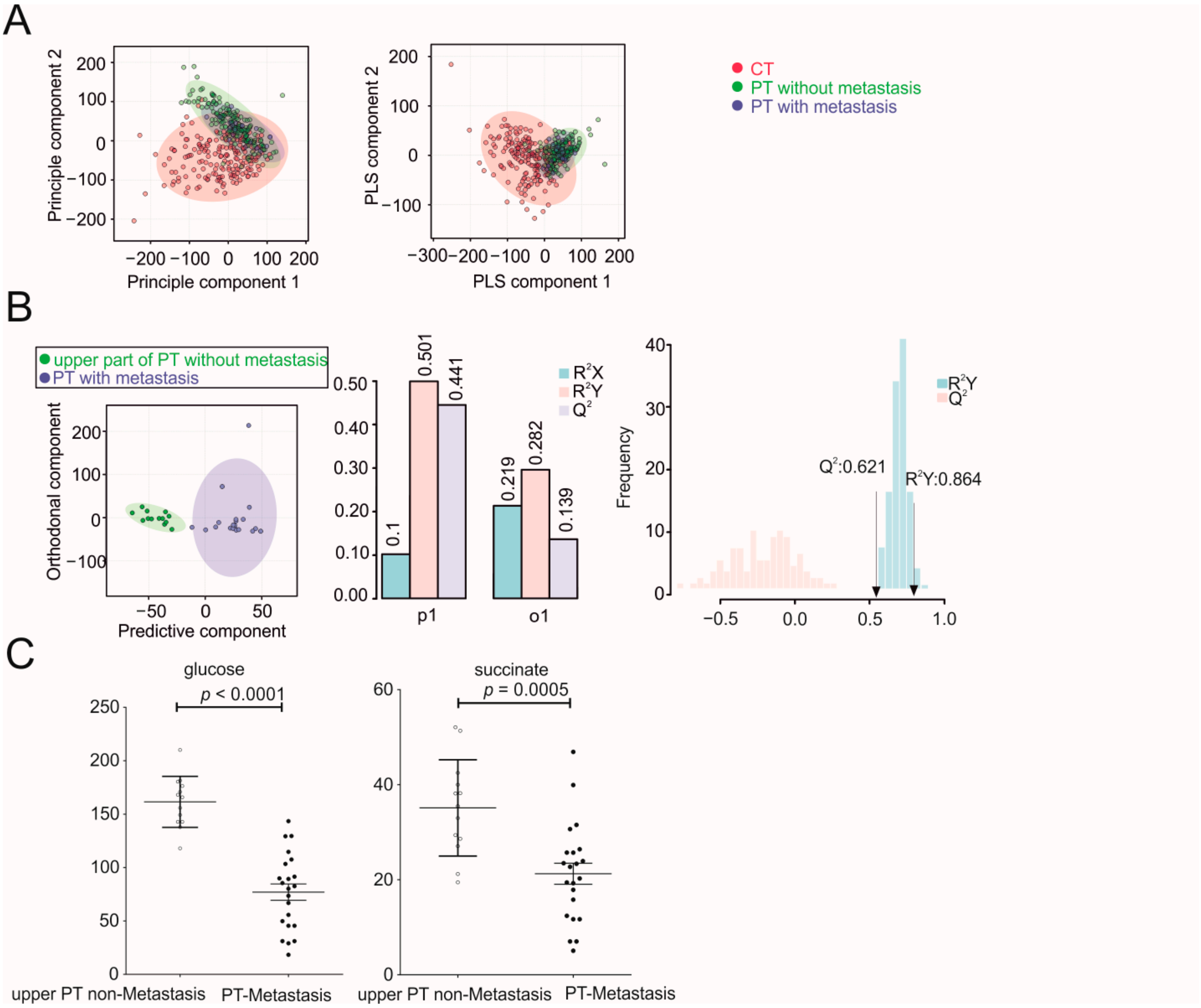

Figure S2. NMR metabolomics analysis of metastasis. (**A**) PCA and PLS-DA plots of tissue samples separated according to metastasis. (**B)** O-PLS-DA plot of peritumoral tissue samples with vs. without metastasis, including and cross validation. (**C**) Statistical analysis of individual metabolites in tissue samples.

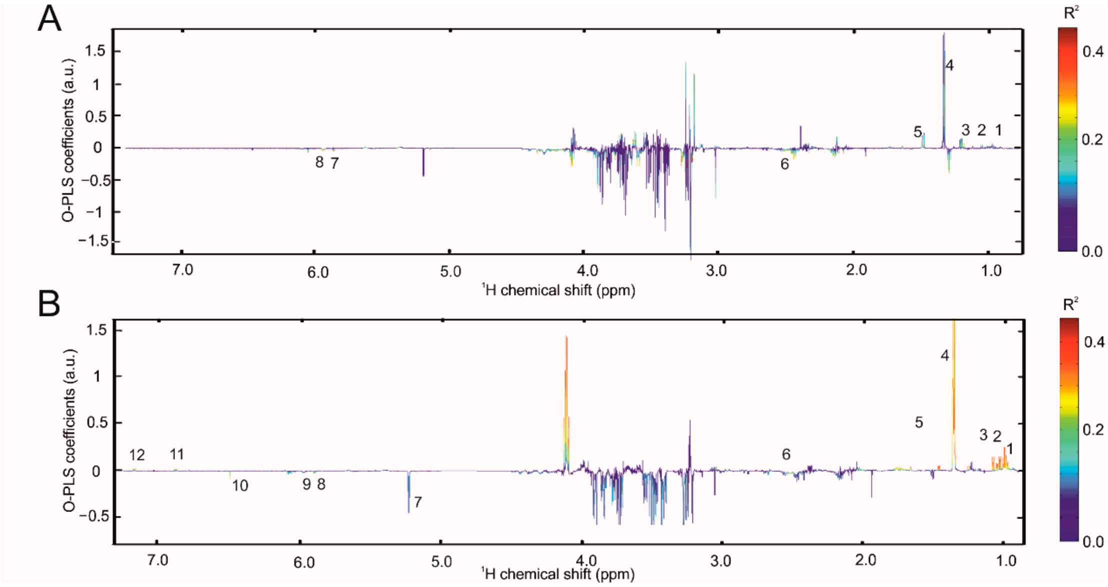

Figure S3. NMR metabolomics analysis of peritumoral and cancer tissue samples at different stages (grade IV vs. grade I). (**A**) The reduced NMR spectrum reveals altered components in normalized peritumoral tissue samples. Positive covariance corresponds to component present at increased concentrations, whereas negative covariance corresponds to decreased component concentration. Predictivity of the model is represented by R^2. 1…leucine, 2…isoleucine, 3…valine,

4…lactate, 5…lysine, 6…glutamine, 7…uridine, 8…UDP-sugars. (**B**) The reduced NMR spectrum reveals altered components in normalized cancer tissue samples. Positive covariance corresponds to component present at increased concentrations, whereas negative covariance corresponds to decreased component concentration. Predictivity of the model is represented by R^2. 1…leucine, 2…isoleucine, 3…valine, 4…lactate, 5…lysine, 6…glutamine, 7…glucose, 8…uridine, 9…UDP-sugars, 10…fumarate, 11…tyrosine, 12…phenylalanine.

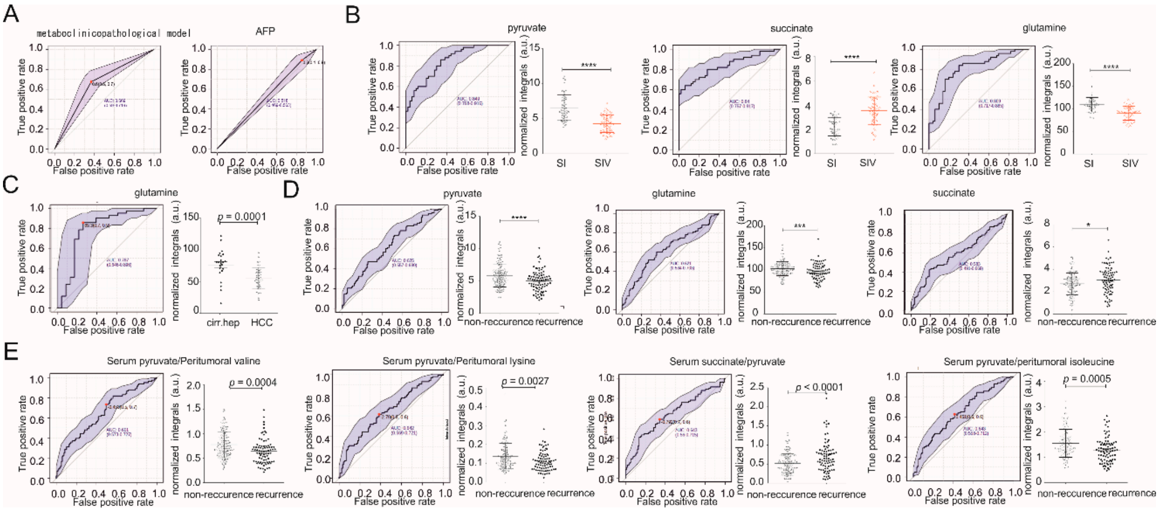

Figure S4. ROC analysis of AFP, metaboclinicopathological model and altered metabolites. (**A**) ROC curve of metaboclinicopathological model (high vs. low risk) and AFP (positive vs. negative) in HCC patients with recurrence versus non-recurrence. Absolute integrals were used to calculate ROC curves for distinct metabolites and to assess the prognostic value of the distinct metabolites of serum for tumor size (**B**), HCC (European cohort) (**C**) and recurrence (**D**). (**E**) Combination of all metabolites (serum and tissues) for ROC analysis. Statistically significant differences between groups (Student's t-test) are indicated by *p*-values of < 0.05 (*), < 0.01 (**), < 0.001 (***) or < 0.0001 (****).

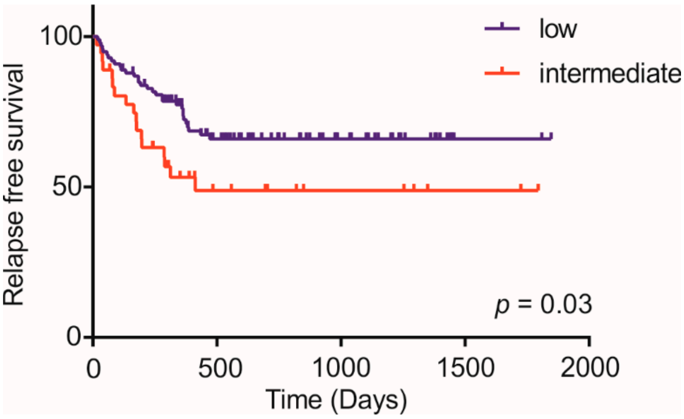

Figure S5. Recurrence-free survival (RFS) according to risk groups defined by Early Recurrence After Surgery for Liver tumor (ERASL)-pre model.

Table S1. Patient demographics and clinical characteristics (Chinese cohort).

Patient Variables	All (*N* = 200)	Grade I (*N* = 50)	Grade II (*N* = 50)	Grade III (*N* = 50)	Grade IV (*N* = 50)
**Age (years)**	54 (23–77)	64 (37–77)	56 (29–75)	54 (23–72)	52 (24–77)
**Female, N (%)**	40 (20%)	11 (22%)	8 (16%)	10 (20%)	11 (22%)
**AFP[a]**					
>10	126	27	25	35	39
≤10	66	21	24	13	8
**ALT[b] U/L**					
>50	38	10	10	18	0
≤50	162	40	40	32	50
**Albumin (g/mL)**	38.83 ± 5.11	38.98 ± 4.36	38.30 ± 7.13	39.12 ± 4.26	38.92 ± 4.21
**Bilirubin (mol/L)**	18.32 ± 14.36	17.58 ± 7.31	22.46 ± 26.90	15.91 ± 5.90	17.42 ± 8.22
**Hepatitis B**	174	45	40	48	41
**Diameter of tumor**	6.41 ± 4.47	2.04 ± 0.53	3.64 ± 0.53	7.15 ± 1.37	12.81 ± 3.02
**Cirrhosis**					
N	70	7	11	20	32
Nodular	130	43	39	30	18
**MVI[b]**					
**MV0**	119	40	35	25	19
**MV1**	37	7	11	12	7
**MV2**	44	3	4	13	24

[a] AFP, alpha-fetoprotein, AFP value > 10 is positive through the manufacturer's introduction (8 patients did not receive AFP measurement). [b]ALT, Alanine aminotransferase. MVI, microvascular invasion, MV0 means the number of MV is 0, MV1 means the number of MV = 1–5, MV2 shows the number of MV > 5.

Table S2. Univariate and multivariate analysis of pre-operative factors.

Variables	Recurrence			
	Univariate Analysis		Multivariate Analysis	
	HR (95%)	*p* Value	HR (95%)	*p* Value
Gender (female vs. male)	1.576 (0.663–3.743)	0.303		
Age (≥55 vs. <55)	0.657 (0.355–1.216)	0.181		
Cirrhosis (yes vs. no)	0.593 (0.324–1.085)	0.09		
Tumor number (single vs. multiple)	2.045 (1.396–2.998)	**0.000**	2.242 (1.540–3.264)	**0.000**
AFP (≥10 vs. <10)	1.315 (0.691–2.504)	0.405		
HBV (yes vs. no)	0.903 (0.322–2.533)	0.847		
DCP (>40 vs. ≤40)	1.610 (0.790–3.283)	0.190		
ϒ-GT (>60 vs. ≤60)	2.501 (1.345–4.650)	**0.004**		
LDH (>245 vs. ≤245)	1.866 (0.915–3.803)	0.086		
ALP (>125 vs. ≤125)	2.285 (1.163–4.491)	**0.016**		
PT (>14.5 vs. ≤14.5)	0.543 (0.193–1.527)	0.247		
ALB (>34 vs. ≤34)	1.057 (0.376–2.967)	0.916		
ALT (≥50 vs. <50)	2.003 (1.060–3.785)	**0.032**	2.552 (1.291–5.046)	**0.007**
AST (≥40 vs. <40)	1.898 (1.028–3.507)	**0.041**		
TBIL (≥26 vs. <26)	0.650 (0.157–2.696)	0.553		
TG (>1.7 vs. ≤1.7)	1.250 (0.384–4.068)	0.771		
Platelet (>125 vs. ≤125)	1.010 (0.524–1.946)	0.976		
No. of WBC (>9.5 vs. ≤9.5)	0.858 (0.207–3.557)	0.833		
Serum succinate	1.393 (1.009–1.921)	**0.044**		
Serum glucose	0.987 (0.976–0.999)	**0.029**		
Serum succinate/serum pyruvate	3.941 (1.219–12.748)	**0.022**	4.572 (1.360–15.372)	**0.014**

AFP, alpha-fetoprotein; ALB, albumin; PT, prothrombin time; ALT, alanine aminotransferase; AST, aspartate aminotransferase; TBIL, total bilirubin; TG, triglyceride; WBC, white blood cell; DCP, abnormal prothrombin; ϒ-GT, glutamyltranspeptidase; LDH, lactate dehydrogenase; ALP, alkaline phosphatase; Significant *p*-values are indicated in bold (significance considered $p < 0.05$).

Table S3. Univariate analysis of other factors.

Variables	Recurrence	
	Univariate Analysis	
	HR (95%)	*p* Value
Tumor size	1.120 (1.052–1.193)	**0.000**
Tumor encapsulation (present vs absent)	0.424 (0.244–0.736)	**0.002**
Microvascular invasion (yes vs no)	1.316 (0.715–2.420)	0.377
TNM stage	1.524 (1.102–2.106)	**0.011**
peritumoral isoleucine	1.450 (1.075–1.957)	**0.015**
peritumoral glucose	0.995 (0.991–1.000)	**0.042**
peritumoral valine	1.308 (1.110–1.541)	**0.001**
peritumoral UDP-sugars	0.894 (0.818–0.976)	**0.013**
cancers lactate	1.000 (1.000–1.000)	**0.005**
peritumoral lactate	1.000 (1.000–1.000)	**0.023**
serum pyruvate/peritumoral valine	0.177 (0.049–0.637)	**0.008**
serum pyruvate/peritumoral isoleucine	0.445 (0.239–0.829)	**0.011**
serum pyruvate/tumoral lactate	0.000 (0.000–0.000)	**0.033**
serum pyruvate/peritumoral leucine	0.000 (0.000–0.193)	**0.02**

Significant *p*-values are indicated in bold (significance considered $p < 0.05$).

Table S4. Multivariable Cox regression analyses of prognostic factors in the derivation and validation cohort without metabolic factors.

Variable	Derivation Group			Validation Group		
	**Hazard Ratio (95% CI)**	**β-Estimate (95% CI)**	***p* Value**	**Hazard Ratio (95% CI)**	**β-Estimate (95% CI)**	***p* Value**
ALT						
<50	Ref	Ref		Ref	Ref	
>50	2.424 (1.230, 4.778)	0.8850 (0.207, 1.564)	0.011	1.040 (0.546, 1.982)	0.039 (− 0.606, 0.684)	0.905
Tumor number	2.241 (1.519, 3.305)	0.807 (0.418, 1.195)	<0.0001	2.074 (1.509, 2.850)	0.729 (0.411, 1.047)	<0.0001

Model score = tumor number * 0.807 + ALT * 0.885; PI = e^ model score/(1 + e^model score);.Cut-off to generate the risk groups: PI ≤ 0.691 (low), PI > 0.691 (high).
